# Implementation of flipped classroom combined with problem-based learning: an approach to promote learning about hyperthyroidism in the endocrinology internship

**DOI:** 10.1186/s12909-019-1714-8

**Published:** 2019-07-30

**Authors:** Xiaolei Hu, Hengyan Zhang, Yuan Song, Chenchen Wu, Qingqing Yang, Zhaoming Shi, Xiaomei Zhang, Weidong Chen

**Affiliations:** 1 Department of Endocrinology, The First Affiliated Hospital of Bengbu Medical College, Bengbu, Anhui China; 2 Department of Diagnostics, Bengbu Medical College, Bengbu, Anhui China; 3 Clinical School of Medicine, Bengbu Medical College, Bengbu, Anhui China

**Keywords:** Flipped classroom, Problem-based learning, Hyperthyroidism, Endocrinology, Internship

## Abstract

**Background:**

With the development of medicine, new teaching methods, such as flipped classroom and problem-based learning (PBL), have received much attention in medical education. However, the implementation of flipped classroom combined with PBL in endocrinology education has not been well investigated. Considering that both two teaching methods may complement each other, therefore, the aim of this study was to evaluate students’ learning effectiveness acceptability of the pedagogy between traditional lecture-based teaching methods and the combination of flipped classrooms with PBL teaching methods in the endocrinology internship.

**Methods:**

74 fourth-year medical students at the Bengbu Medical College were enrolled in the endocrinology internship. Hyperthyroidism was chosen for the content of this study. The participants were randomly allocated into either the combination group of flipped classroom with PBL (CG) or the traditional lecture-based classroom group (TG). Both a pre-quiz and a post-quiz were conducted before and after the classes, respectively. All questions in the quizzes were classified into two aspects, basic theoretical knowledge and clinical case analyses based on the Bloom’s Taxonomy. The scores were compared and students were required to complete the questionnaire to evaluate their perceptions and experience.

**Results:**

The mean post-quiz scores of both the TG and the CG were higher than those of the pre-quiz. Additionally, the post-quiz showed that students in the CG had significantly higher scores in the TG. Further analysis found that after class, only the difference in clinical case analysis between CG and TG was significant. The scores of all items in the questionnaires were higher in the CG than in the TG. More students agreed that the combined teaching method could help to improve their performance, at the same time, it could increase their workload.

**Conclusions:**

The combination of the flipped classroom and PBL teaching approach could be a better option over the traditional lecture-based classroom in the teaching of hyperthyroidism during endocrinology internship, although it can increase students’ workload. To be widely accepted and implemented, further optimizations are required.

## Background

With the development of society, the demands for medical and health services have increased, which directly affect the professional level and service quality of doctors. Therefore, medical education should focus on training the medical students to have appropriate professional attitudes, acquire appropriate clinical skills and prepare them to handle real-life situations [1].

In recent years, new medical teaching methods have emerged that focus on strengthening practical teaching, improving medical students’ comprehensive thinking ability and solving clinical practical problems. Problem-based learning (PBL), presented as a new teaching method for the first time at McMaster University in Canada in the 1960s, has long been accepted as a cornerstone in many medical education curricula worldwide [2]. The PBL, similar to case-based learning but different in many ways including goals, focus, roles and amount of cases, is a student-centric learning procedure in which a teacher only acts as a moderator, using a problem as a starting point for learning. [3, 4] In PBL, students are divided into several groups and conduct research on the topic of interest, and the questions are then discussed [5]. It is undeniable that PBL is one of the proposed ways to improve the correlation between the educational and clinical performance of students. Many studies and our previous study have found that the implementation of PBL increased the clinical practice skills of residents, improved student satisfaction with teaching and enhanced the ability of students to independently conduct a study using problem-solving and analytical skills [5–9]. Although PBL is growing worldwide, this model is still controversial [10, 11]. There are two disadvantages of PBL. For example, 1) it requires students to spend a great deal of time preparing for problems before class, which is very difficult for medical students who have just been introduced to busy clinical work, and 2) students have limited access to learning materials, which reduces the effect of teaching.

Currently, with the development of information and communication technologies, personal computers and mobile information platforms such as smartphones and tablet computers have resulted in e-learning becoming part of higher education in many fields [12, 13]. The flipped classroom, also called inverted classroom [14], is a pedagogical model, first described by Lage, Platt and Treglia in 2000 and then popularized by Bergmann and Sams in 2012 [15, 16], and now, it has received much attention in medical education [17]. The flipped classroom is one of the blended learning methodologies that combines e-learning and face-to-face classroom technique, which is intended to improve the efficacy of classroom learning by allowing students to control the timing and pace of their online learning and maximize their opportunity for active learning by engaging in class discussions and collaborative exercises in the company of peers and instructors [18]. Thus, to some extent, the flipped classroom can make up for the disadvantages of PBL. Several recent systematic reviews have found that compared to traditional lecture-based classrooms, students in flipped classrooms consistently report greater satisfaction and more positive academic outcomes, motivation and engagement [17, 19]. However, to our knowledge, very few literatures involved in the teaching method of combination the flipped classroom with PBL in medical education, and there is even no research in endocrinology, which is one of the most important subjects in internal medicine. Considering the differences between different disciplines, to evaluate the effectiveness and acceptability of the pedagogy that combines the flipped classroom with PBL in endocrinology internship teaching, we performed the current study involving 74 fourth-year medical students. We compared the students’ performance, experience and competence in learning about hyperthyroidism between this combined pedagogy and the traditional lecture-based classroom. The purpose of this study is to provide guidance for instructors who are considering using this combined pedagogy in medical education.

## Methods

### Participants

A total of 74 fourth-year medical students majoring in clinical medicine at the Bengbu Medical College were enrolled in the endocrinology internship at the First Affiliated Hospital of Bengbu Medical College. They had attended all the endocrinology lessons provided in Bengbu Medical College by the same instructors. These participants were randomly allocated into either the combination group of the flipped classroom with problem-based learning (CG, *n* = 37) or the traditional lecture-based classroom group (TG, *n* = 37). All students were unaware of their group assignments before the internship. Both groups were supervised by a teaching group composed of one instructor and three assistants who had worked in the Department of Endocrinology. Informed consent was obtained from all subjects, and the research was approved by the Institutional Review Board and Ethics Committee of the First Affiliated Hospital of Bengbu Medical College.

### Study design

We chose the chapter of hyperthyroidism to apply the combination approach for this study because the clinical signs and symptoms in hyperthyroidism are easier for students to observe and understand.

The CG proceeded as follows: before the class, the instructor prepared the relevant lecture videos and supplementary materials for the course. Each student needed to view them on his or her own time. During the class, prior to any classroom activities, the students were asked to complete a pre-quiz with thirty-two multiple-choice questions about hyperthyroidism. The class session started with a brief introduction of the topic and class agenda by the instructor. Then, a patient case with slides and several relevant questions were presented by the instructor as follows: “(1) What is the pathogenesis of hyperthyroidism? (2) How does one distinguish between hyperthyroidism and thyrotoxicosis? (3) What are the clinical manifestations of hyperthyroidism? (4) What are the diagnostic criteria for hyperthyroidism? (5) What are the treatment methods of hyperthyroidism and how are they chosen?” Next, the students entered into small-group discussion format, with the instructor guiding the discussion. After that, a student representative from each small group made a presentation to review the main points from the lecture, proposed their answers to the questions and asked the unsolved questions from his or her small group. Finally, the instructor summarized for the class and went over the tough questions raised during discussions. At the end of these classroom activities, students were asked to complete a post-quiz with the same questions about hyperthyroidism as in the pre-quiz, as well as a post-survey with eleven questions on their perception of and experience with the combined classroom.

The TG followed the procedure described in our previous study [9]. In brief, prior to the lecture, students were only told to prepare for class, instead of watching the videos or reading the supplementary materials; then, they took a pre-quiz with the same thirty-two multiple-choice questions as the CG. These students accepted the equivalent content in the traditional teaching method, and a declarative explanation of the theoretical knowledge was provided by the instructor according to the teaching outline, instead of dividing the class into small groups to discuss the cases. That is, the instructor’s teaching was the predominant approach used. After the class, the instructor assigned homework involving the same questions discussed in the CG and the same post-survey as the CG.

**Fig. 1:**
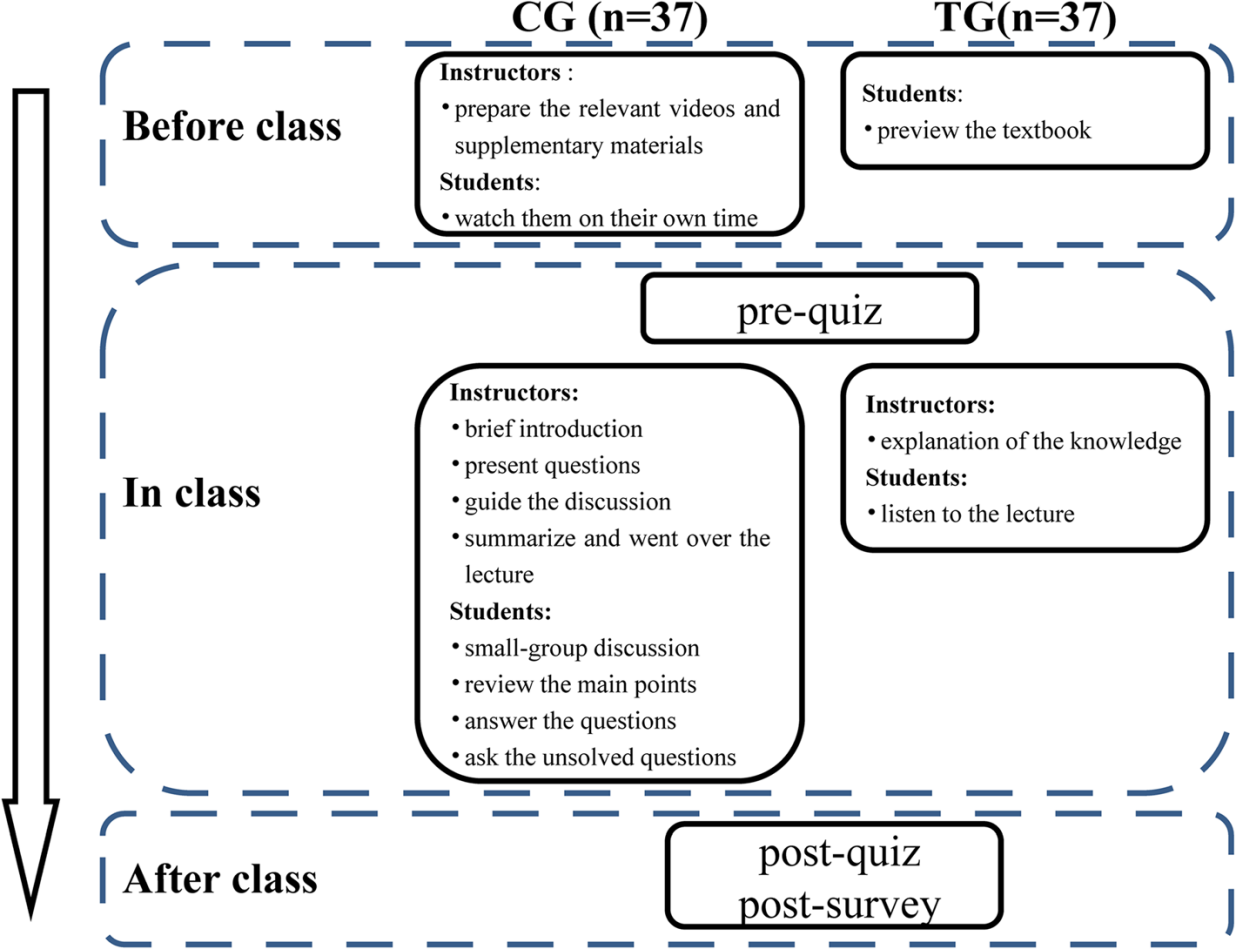
A graphical overview of the study design. TG: traditional lecture-based classroom group, CG: combination group of flipped classroom with problem-based learning

All the students were also given a consent form and were informed that taking the quizzes and completing the surveys were voluntary. As numbers were used in the quizzes and surveys instead of real names, it would have no positive or negative effect on students’ grades or performance in the class. The quizzes and surveys were taken and filled out without any discussion or assistance from peers or faculty. The graphical overview of the study design was shown in Fig. 1.

### Data evaluation and statistical analysis

To evaluate students’ understanding of the hyperthyroidism course, both a pre-quiz and a post-quiz were conducted before and after the classes, respectively. These two quizzes had the same multiple-choice questions, which included two aspects, basic theoretical knowledge (40 points) and clinical case analysis (60 points). All questions in the quizzes were based on the Bloom’s Taxonomy, which was categorized cognitive activities into six hierarchical levels, including remember, understand, apply, analyze, evaluate and create [20] . The “remember” and “understand” categories were collapsed into one category called “basic theoretical knowledge”. Items in any of the other categories were considered “clinical case analyses”. [21] We calculated the total scores of the questions for each student, and then, the scores were compared between the two groups by an independent samples t-test and between before and after the class by a paired sample t-test. After the class, students from both groups were required to complete the same anonymous questionnaire to evaluate their perceptions and experience. The evaluation criteria were based on our previous study [9]: according to the degree of improvement, scores were divided into 5 grades, from 1 point (poor) to 5 points (excellent). The scores between two groups were analyzed using an independent samples t-test. Furthermore, a score ≥ 4 was defined as satisfactory. The chi square test was used to compare the rates. In addition, the time spent on task in both groups were measured, specifically, the time spent on preparation before class in the CG includes time for students to watch relevant lecture videos and materials, as well as search supplement materials on the Internet, and the time in the TG includes time for students to preview the textbook. All statistical analyses were performed using the SPSS 22.0 version (Chicago, USA). Alpha was set at 0.05, and *p*-values of less than 0.05 were considered significant.

## Results

### Demographic information of participants

A total of 74 students were enrolled in the study, including 37 students assigned to the TG and 37 students assigned to the CG. The gender ratio and ages for the two groups were comparable (Table 1).

**Table 1:** Demographic information of participants

	TG	CG	Statistics	*P* value
Total	37	37		
Gender				0.816^a^
Male	19 (51.4%)	18 (48.6%)	χ2 = 0.054	
Female	18 (48.6%)	19 (51.4%)		
Age(y)	22.4 ± 0.9	22.1 ± 1.0	t = 0.874	0.389^b^

TG: traditional lecture-based classroom group, CG: combination group of flipped classroom with problem-based learning

^a^The two groups were compared using the chi-square test

^b^The two groups were compared using independent samples t test

### Students’ scores of quizzes

**Fig. 2:**
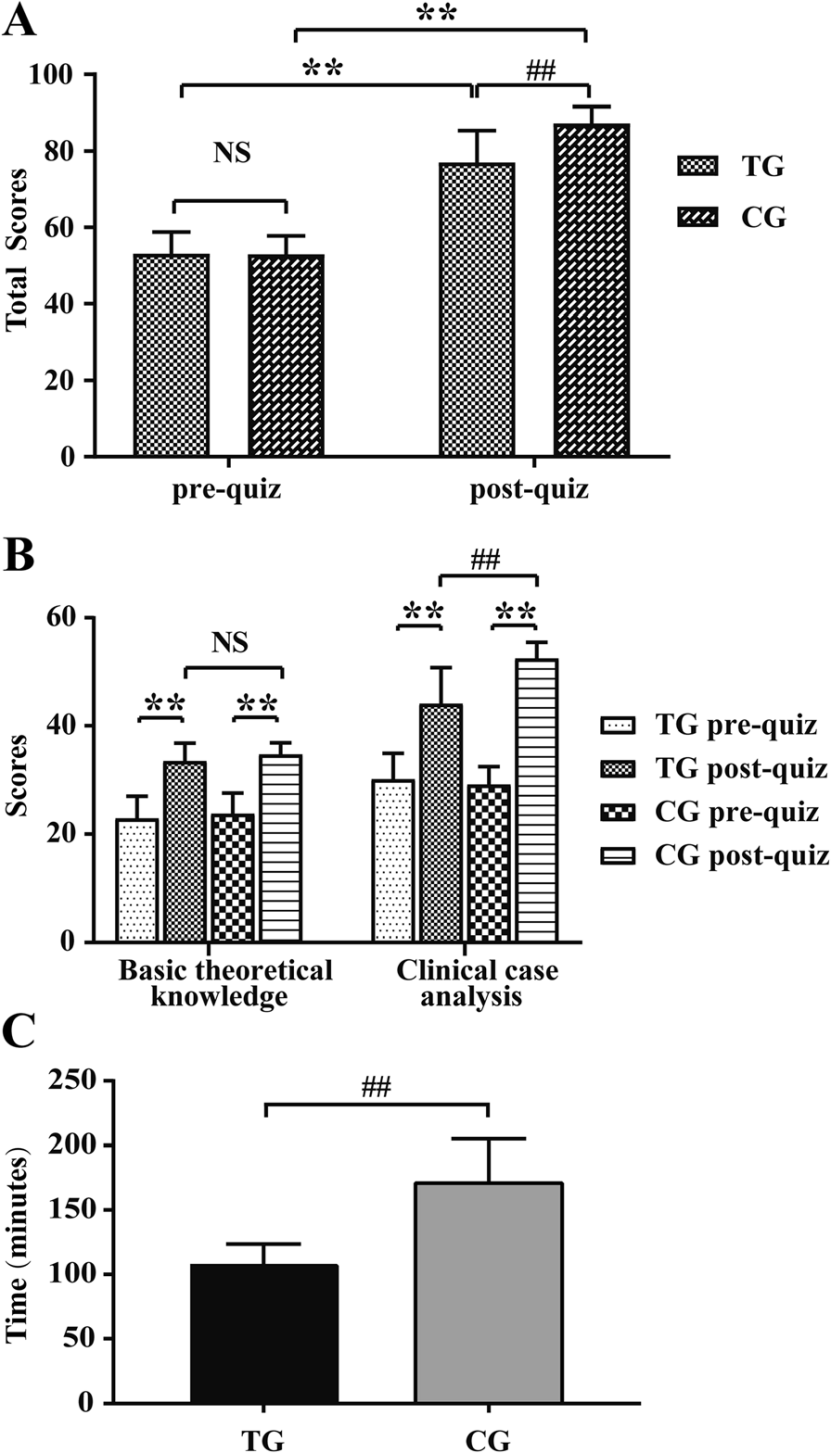
Mean scores of pre- and post-quiz in TG (*N* = 37) and CG (N = 37). (**a**). Comparison of mean pre- and post-quiz total score between TG and CG. (**b**). Comparison of mean pre- and post-quiz score on basic theoretical knowledge and clinical case analysis between TG and CG, respectively. TG: traditional lecture-based classroom group, CG: combination group of flipped classroom with problem-based learning. Data are presented as the means ± standard deviation (SD); ** *p* < 0.001 post-quiz vs. pre-quiz, ^##^ *p* < 0.001 CG vs. TG, and NS: no significant difference

In the TG, the mean pre-quiz score was 53.1, and in the CG, the mean pre-quiz score was 53.0. There were no significant differences between the two groups (*p* > 0.05) (Fig. 2a), indicating that the baseline between the two groups was comparable. After the classes, in the TG, the mean post-quiz score increased by 24.0 to 77.1 (*p* < 0.001); in the CG, the mean post-quiz score increased by 34.3 to 87.3 (*p* < 0.001) (Fig. 2a). That is, students’ quiz scores improved in both groups and the enhancement in the two groups was also significant. Additionally, the post-quiz showed that students in the CG had significantly higher scores than those in the TG (*p* < 0.001) (Fig. 2a). Further analysis found that in both basic theoretical knowledge and clinical case analysis, compared with the pre-quiz, the post-quiz scores were significantly improved, regardless of which teaching method was adopted (*p* < 0.001) (Fig. 2b). Furthermore, there was no significant difference in basic theoretical knowledge between the TG and CG after classes (*p* > 0.05). However, the most improvement was observed in the CG regarding analyzing clinical cases (p < 0.001) (Fig. 2b).

### Students’ perspectives, self-perceived competence and satisfaction survey

Next, students’ perspectives and self-perceived competence were compared after taking the TG and CG, respectively. As shown in Table 2, the scores of all items in the questionnaires were higher in the CG than in the TG (all *p* < 0.05). We further classified the scores, of which greater than or equal to four points was defined as satisfactory. Compared with the TG, more students agreed that the CG could help improve their learning motivation (*P* < 0.001), prepare them for the examination (*P* < 0.001), enhance student-teacher interaction (*P* < 0.001), and promote their multi-abilities, including communication ability (P < 0.001), clinical thinking ability (*P* < 0.001), self-learning ability (*P* < 0.001), teamwork ability (*P* < 0.001), knowledge acquisition ability (*P* < 0.01) and presentation and expression ability (*P* < 0.001) (Table 3). However, the CG was not inferior to the TG in helping students to understand the course (*P* = 0.155). Instead, the students in the CG spent significantly more time preparing for class than those in the TG (108 ± 15 min vs. 172 ± 33 min) (*P* < 0.001) (Fig. 2c, Tables 2 and 3).

**Table 2:** Comparison of students’ perspectives and self-perceived competence between TG and CG

Items	TG	CG	*P* value^a^
Learning motivation	3.5 ± 0.6	4.4 ± 0.6	0.000
Understanding the course	4.0 ± 0.6	4.2 ± 0.6	0.026
Student-teacher interaction	3.0 ± 0.5	4.5 ± 0.6	0.000
Occupies too much spare time	3.3 ± 0.5	4.2 ± 0.6	0.000
Final examination	3.5 ± 0.7	4.2 ± 0.6	0.000
Communication ability	3.5 ± 0.6	4.4 ± 0.7	0.000
Clinical thinking ability	3.4 ± 0.6	4.3 ± 0.5	0.000
Self-learning ability	3.1 ± 0.8	4.3 ± 0.6	0.000
Teamwork ability	2.9 ± 0.6	4.6 ± 0.6	0.000
Knowledge acquisition ability	3.5 ± 0.6	4.5 ± 0.7	0.000
Presentation and expression ability	3.5 ± 0.6	4.3 ± 0.5	0.000

*TG* traditional lecture-based classroom group, *CG* combination group of flipped classroom with problem-based learning

According to the degree of improvement, scores were divided into 5 grades, from 1 point (poor) to 5 points (excellent)

^a^The two groups were compared using independent samples t test

**Table 3:** Comparison of students’ satisfaction survey between TG and CG

Items	Degree of Satisfaction		χ2	*P* value^a^
	TG	CG		
Learning motivation	51.4%	97.3%	18.128	0.000
Understanding the course	81.1%	94.6%	2.024	0.155
Student-teacher interaction	10.8%	97.3%	52.290	0.000
Occupies too much spare time	35.1%	89.2%	20.741	0.000
Final examination	54.1%	91.9%	11.580	0.000
Communication ability	56.8%	89.1%	8.291	0.000
Clinical thinking ability	45.9%	97.3%	21.542	0.000
Self-learning ability	32.4%	91.9%	25.337	0.000
Teamwork ability	13.5%	97.3%	49.224	0.000
Knowledge acquisition ability	54.1%	91.9%	9.574	0.002
Presentation and expression ability	56.8%	97.3%	14.968	0.000

*TG* traditional lecture-based classroom group, *CG* combination group of flipped classroom with problem-based learning

According to the degree of improvement, scores were divided into 5 grades, from 1 point (poor) to 5 points (excellent). The scores were classified, of which greater than or equal to four points was defined as satisfactory

^a^The two groups were compared using the chi-square test

## Discussion

Currently, traditional lectures do not align well with modern learning theory and do not take full advantage of our current knowledge of how people learn [22]. With technological advancements and the explosion of innovative approaches to education in general, there are many alternative (and arguably superior) approaches to the traditional didactic lecture, including the flipped classroom and problem-based learning [22]. Most previous studies focus on either of them separately, however, these two teaching methods both have several disadvantages. For example, in PBL, some students complained about the time requirements and the lack of suitable learning materials [23, 24], and in the flipped classroom, some students suggested that reduced instructor contact could negatively affect them and that learning effects would not be improved without increased direction and support from faculty [22, 24–26]. Therefore, combining the flipped classroom and PBL methods may allow for them to complement each other: on the one hand, pre-class teaching materials in flipped classrooms provided by instructors can save students time for preparation and lay a good foundation for the development of teaching in PBL; on the other hand, the small-group, teacher-oversight module in PBL can also make teaching more efficient.

Hyperthyroidism is one of the most important chapters in endocrinology, thus, it is necessary to find an effective teaching method. We chose hyperthyroidism as the topic for our study because it has many typical clinical symptoms and signs and is not overly complicated to prepare at home without an instructor; a hands-on approach with case studies from our wards could be used for class exercises.

Our study was a further investigation into the effectiveness and suitability of the combination of the flipped classroom and PBL method in a clinical education setting of hyperthyroidism, which was developed based on our previous pilot study [9]. Thus far, this combined method has not been used in endocrinology education, especially in hyperthyroidism education. In this study, regardless of which teaching method was adopted, the mean total scores improved significantly after the classes. Compared with the TG, the total scores in the CG were higher (Fig. 2a). Our findings were consistent with the results from several medical teaching studies that demonstrated improvement in examination scores or final grades associated with the flipped classroom combined with PBL teaching mode [27–29]. These findings have shown that this combined teaching method is more effective in helping students master and apply hyperthyroidism knowledge.

Notably, we further analyzed the types of quizzes and found that the dominant improvement in total scores from pre- to post-quiz was the clinical case analysis because there was no significant difference in basic theoretical knowledge between the two groups (Fig. 2a). Two factors may contribute to these results. First, in our study, all questions of the quizzes were based on the Bloom’s Taxonomy, which was widely used in educational research to stratify learning activities into different cognitive levels that range from basic recall to higher educational objectives, including remember, understand, apply, analyze, evaluate and create [20]. The “basic theoretical knowledge” consists of “remember” and “understand” categories, so it is considered a “low” level cognition [21]. Moreover, it comes from the same textbook, and it is all rote learning, thus, little difference was found. Second, the clinical case analysis, involved in the other four categories, primarily investigates the ability of students to use basic theoretical knowledge to find and solve problems. Both the flipped classroom and PBL are problem-solving teaching methods, emphasizing self-study or self-directed learning [22] that belongs to a “high” level cognition [21]. As a result, the combination of these two methods can improve the understanding of theoretical knowledge and strengthen practical skills.

In addition, we compared students’ perspectives and self-perceived competence between the two groups in our study. Compared with the TG, the scores in the CG increased according to the post-class surveys (Table 2). Overall, all the students in the CG were satisfied with this new teaching method (Table 3), for example, 1) students in the CG felt more motivated to learn when compared to those in the TG; 2) students considered the combined teaching approach to be more helpful for final examination and student-teacher interaction; and 3) students in the CG felt more improvement in their multi-abilities, such as communication, clinical thinking, self-learning, teamwork, knowledge acquisition, presentation and expression. Similar to our findings, previous studies have shown that the combined approach improves students’ skills and competence in many medical fields, including preventive medicine [30], urology [29], physiology [24] and biochemistry and molecular biology [28]. There are several possible explanations. First, in the CG, the flipped classroom approach offers personalized study, so students in the flipped classroom have more freedom and flexibility for self-paced learning, providing students an opportunity to use their time more efficiently [31, 32]. Second, in the CG, the PBL approach encourages group discussions and presentations, which help to retain information and improve cognitive skills [22, 33]. Finally, compared to the traditional teaching method, which focuses on how much knowledge can be absorbed in class by the students through reading and listening (emphasis on input), both flipped classroom and PBL encourage students to verbalize what they learn and to exchange ideas through discussion or debate (emphasis on output) [31, 33]. As a result, it is no wonder that the combined methodology can enhance students’ perspectives and self-perceived competence. Consistent with our findings, previous studies have shown that the combined approach improves students’ various abilities [27–30].

It is also worth noting that students in the CG spent much more spare time preparing for class than those in the TG (Table 2). These results suggest that although the combined approach can improve student performance, at the same time, it can increase their workload. As a popular saying goes, every coin has two sides. On one hand, spending more time means students can get more knowledge and deeper understanding. It might in part explain why students in the CG have higher scores, especially in clinical case analysis which requires students understand the content in-depth. On the other hand, spending more time on study means it will occupy too much spare time. This may be the reason why there is no difference in student satisfaction with “understanding the course” between the two groups (Table 3) (the burden may compromise the satisfaction). Interestingly, a recent study found that more students agreed that the flipped classroom methodology could help them understand the course material [31]. This discrepancy between studies may be attributed to the differences in each study, such as teaching method, students’ knowledge background and course selection. Additionally, another well-designed study, which focused on the details of effective implementation of the flipped classroom, indicated that the format, quantity, quality of preparatory materials and the time spent on preparation before class were all important factors for effectiveness and efficiency in implementation of the flipped classroom. [34]

### Limitations

There are two limitations to this study. One is that the study took place in only one clinical department of a medical college, so its results may not be generalizable to students in other colleges. Another limitation is that we assessed only the short-term results of the combined method. Therefore, further studies are required to investigate whether the benefits of this combined method extend to the long term as well.

## Conclusions

In summary, the findings from our study suggest that a combination of the flipped classroom and PBL teaching approach may help to improve student performance and enhance their multiple clinical skills and abilities when learning about hyperthyroidism during the endocrinology internship. Further studies are needed to evaluate how to optimize course design and course administration to allow for the broader implementation of the combined model into other curricula.

## Data Availability

Not applicable.

## Abbreviations

CG: Combination group of flipped classroom with PBL
PBL: Problem-based learning
TG: Traditional lecture-based classroom group
